# Australasian Science Association

**Published:** 1888-10-27

**Authors:** 


### Australasian Science Association.

The meetings held at Sydney, New South Wales, at the end of August, of the Australasian Association for the Advancement of Science were most interesting. In 1884 the British Association visited Canada, and immediately Australia was fired with ambition to also entertain that distinguished society. On calmer reflection, however, the notion was found too extravagant to be carried out, and Professor Liversidge, of Sydney University, came forward with the more feasible suggestion of establishing an association of their own. The meetings in August were the outcome of this sensible project, and were extremely well attended. Amongst the many papers read, the following dealt chiefly with subjects interesting to our readers.

Dr. Bancroft, of Brisbane, delivered a valuable presidential address on the health conditions of Australia. After commenting on the difficulties of getting people to realise the importance of sanitary science, he proceeded to discuss, in a scientific and admirably practical spirit, the actual problems in health that have to be faced in Australia. The climate, he said, ought to be favourable to long life, but the startling largeness of the mortality among children under five years old reduces the average lifetime considerably. The first definite problem, then, is to improve the conditions of life for infants, and Dr. Bancroft suggested that as a first step the dwellings ought to be improved in the direction of increased size, and the duplication of the corrugated iron roof, with packing between the two roofs. In the country more care should be exercised in fixing the sites of houses, so that refuse fluids would never be permitted to flow towards the house. With regard to the spread of typhoid fever, Dr. Bancroft opposed the usually-accepted view that typhoid is spread solely by sewage infection of drinking water. He wants typhoid to be put on the same footing in the matter of precautions as other contagious fevers.

Dr. Ashburton Thompson, the chief medical inspector of the Board of Health of New South Wales, described a new method of classifying vaccine scars, and the several degrees of severity shown by post-vaccinal small-pox. He had devised the method in order that the effects of vaccination in avoiding death and disfigurement might be more exactly measured than is possible on the plan of classifying at present generally followed.

Dr. Campbell, of Adelaide, read a paper on "The Deep Drainage System of Adelaide: Its Results and Mistakes." This elicited a brisk discussion, as the knowledge that Melbourne is probably going in for a large sewerage system has stirred up a lively interest in sewage problems all over Australia. Dr. Campbell showed what benefit Adelaide had clearly derived from adopting the underground system, but pointed out that the "boundary traps," which are intended to keep foul gas from welling back from the main sewers to the dwelling pipes, are only a provision for catching the evil thing. The only safe means is to keep continually sweeping the air into the sewers, so that no accumulation of bad gas can occur. The whole point of Dr. Campbell's paper lay in the insistence of a thorough system of continual ventilation.

